# Regulatory Dendritic Cells, T Cell Tolerance, and Dendritic Cell Therapy for Immunologic Disease

**DOI:** 10.3389/fimmu.2021.633436

**Published:** 2021-03-10

**Authors:** Sara Ness, Shiming Lin, John R. Gordon

**Affiliations:** ^1^ Department of Medicine, College of Medicine, University of Saskatchewan, Saskatoon, SK, Canada; ^2^ Division of Respirology, Critical Care and Sleep Medicine, College of Medicine, University of Saskatchewan, Saskatoon, SK, Canada

**Keywords:** tolerance, dendritic cell, regulatory T cell, immunologic disease, mechanism

## Abstract

Dendritic cells (DC) are antigen-presenting cells that can communicate with T cells both directly and indirectly, regulating our adaptive immune responses against environmental and self-antigens. Under some microenvironmental conditions DC develop into anti-inflammatory cells which can induce immunologic tolerance. A substantial body of literature has confirmed that in such settings regulatory DC (DCreg) induce T cell tolerance by suppression of effector T cells as well as by induction of regulatory T cells (Treg). Many *in vitro* studies have been undertaken with human DCreg which, as a surrogate marker of antigen-specific tolerogenic potential, only poorly activate allogeneic T cell responses. Fewer studies have addressed the abilities of, or mechanisms by which these human DCreg suppress autologous effector T cell responses and induce infectious tolerance-promoting Treg responses. Moreover, the agents and properties that render DC as tolerogenic are many and varied, as are the cells’ relative regulatory activities and mechanisms of action. Herein we review the most current human and, where gaps exist, murine DCreg literature that addresses the cellular and molecular biology of these cells. We also address the clinical relevance of human DCreg, highlighting the outcomes of pre-clinical mouse and non-human primate studies and early phase clinical trials that have been undertaken, as well as the impact of innate immune receptors and symbiotic microbial signaling on the immunobiology of DCreg.

## Introduction

In the early 1970s, dendritic cells (DC) were discovered and identified as proficient antigen-presenting cells that were capable of potently activating T cells (1–4). By the early 1990s, DC researchers had begun to uncover the regulatory roles of naturally-occurring DC (5), as well as new ways to convert immature DC into tolerance-promoting antigen-presenting cells (6, 7). However, these experiments were limited due to the small numbers of differentiated DC that can be collected from human or mouse tissues (8, 9), such that it was not until methods were developed to differentiate DC *in vitro* that DC research really became a mainstream sub-discipline in immunology.

As noted above, DC develop from bone marrow progenitor cells that complete their differentiation in the periphery. Classically, they were thought to differentiate into either “migratory” or “tissue resident” cells, although later studies exploring their relative gene expression patterns delineated a more specific classification system based primarily on lineage (10–13). There are now considered to be five categories of DC: plasmacytoid DC (pDC); conventional DC (cDC), including cDC1 and cDC2 (the latter are also called myeloid DC [mDC]); monocyte-derived DC (mo-DC); and Langerhans cells (14–17). Each of these has been identified in multiple mammalian species, including mice, humans, non-human primates (NHP), and pigs (10, 12, 18). The anatomic localization, immunologic functions, and expression of surface markers, secreted mediators and Toll-like receptors [TLR(s)] by these different groups of murine and human DC has recently been reviewed (19).

In general, cDC are well suited for extra- and intracellular pathogen recognition and antigen presentation to naïve CD4^+^ and CD8^+^ T cells, while pDC are more often associated with protective antiviral and systemic autoimmune responses, a consequence of being activated primarily by TLRs that recognize intracellular viral or self-DNA/RNA species (20–24). Langerhans cells are present in the epidermis and have a role in both tolerance and immune priming in that compartment. Mo-DCs differentiate from monocytes recruited during ongoing tissue inflammatory responses [e.g. (25)] and in turn direct the differentiation of CD4^+^ T cells to Th1, Th2, or Th17 cells (26, 27). Recent research has revealed that these DC categories are much more fluid than once thought, inasmuch as convergence of differentiation pathways and transitioning between the different types of DC is evident (28–30), making categorization of distinct DC sub-populations an increasingly nebulous concept.

As suggested, in the later 1990s the development of *in vitro* conditions for generating DC from bone marrow and/or peripheral blood progenitors (31, 32) allowed for a surge in research progress. As sentinel antigen-presenting cells DC are well situated immediately adjacent to or integrated into our epithelial cell interfaces with our external environment (e.g., lungs and gut). As steady state cells they are avidly phagocytic, such that they regularly sample their external environment, ingesting and processing all manner of exogenous and endogenous agents, including apoptotic cells, and load the processed antigen peptides onto MHCII molecules for presentation to T cells. DC that are exposed to inflammatory signals during antigen acquisition upregulate their expression of peptide-loaded MHCII, co-stimulatory (e.g., CD40, CD86) and lymph node-homing chemokine receptors, and their expression of inflammatory mediators such as IL-12, while down-regulating their phagocytic activities. They do so while migrating to draining lymph nodes, where they present their processed antigen peptides to T cells in an immunostimulatory context, activating effector T cell (e.g., Th1, Th17) responses (33). If, however, tissue DC acquire otherwise innocuous antigens (e.g., apoptotic cells) in the absence of inflammatory signals, they do not upregulate their antigen-presenting machinery or inflammatory cytokine secretion. As such they provide only low levels of MHCII-bound antigen peptides, co-stimulatory markers and secreted IL-12 signaling to T cells, and thereby induce T cell anergy (34). On the other hand, the DC from mice exposed to innocuous respiratory antigens produce low levels of IL-10 (and less IL-12), while presenting the processed antigens to T cells, and thereby induce Treg responses (35). In the gut, steady state epithelial cells secrete retinoic acid and TGFβ, such that the endogenous gut DC express CD103, TGFβ, and retinoic acid, and thereby induce the differentiation of TGFβ-secreting CD25^+^LAP^+^Foxp3^−^ Th3-type Treg (36–38). As DC research became more accessible it was clear that such regulatory DC (DCreg) could be induced under many different conditions, many of which gave rise to DCreg of a unique phenotype, with differing capacities to and mechanisms by which they regulate immune responses (39). Because of this, harnessing the tolerogenic potential of DCreg for the treatment of disease calls for careful consideration of the optimal type of DCreg for each application.

Naturally-occurring and induced DCreg are able to generate robust antigen-specific tolerance by suppressing other immune cells, including Teff, as well as by inducing the differentiation of CD4^+^ regulatory T cell (Treg) populations. Tolerogenic DC can also directly or indirectly (40) induce regulatory responses among B cells, natural killer cells and CD8^+^ T cells (41–49), but we will focus herein on DCreg-CD4^+^ T cell interactions. Specifically, we will review more recent DCreg research, focusing on the studies that have been conducted since our last review of this topic (50). We will address recent progress with human DCreg, but we will also discuss non-human studies where they may shed light on gaps in our knowledge. Furthermore, we will examine the literature that characterizes such DCreg at the cellular and molecular levels and will address the tolerogenic potential and clinical applicability of these cells.

### DCreg Suppression of Effector T Cell Responses

DCreg-T cell conversations are facilitated by cell surface molecules and secreted mediators that can directly suppress effector T cell (Teff) responses. We have reported that steady-state CD8α^+^ splenic DC from mice can suppress Th2 Teff cell responses through their expression of the tryptophan-catabolizing enzyme indoleamine-2,3-dioxygenase (IDO), but also through expression of IL-10 and TGFβ (51). IDO depletes the local cell environment of tryptophan, an essential amino acid for T cells, and thereby activates the Generalized Controller Non-derepressible-2 Kinase (GCN2) (16, 32, 33), a sensor of cellular amino acid levels, leading to T cell apoptosis (52). In addition, kynurenine break-down products of tryptophan, including 3-hydroxyanthranilic and quinolinic acids, can induce a caspase-8-dependent, but Fas-independent apoptosis of T cells (53). Nevertheless, Fas/FasL signaling can also play a role in DCreg suppression of Teff cells. For example, CD8α^+^ splenic DC (54) and splenic stroma-educated DCreg (55) induce T cell apoptosis in a Fas/FasL-dependent fashion. In the latter case, FasL signaling activates caspases-3 and -8 in T cells to directly activate apoptosis. However, FasL signaling by DCreg also augments IFNγ secretion by T cells, which in turn induces NO production by the DC, and that further augments CD4^+^ T effector cell apoptosis (55).

Many reports have addressed the induction of T cell anergy by IL-10-induced human DCreg which, as semi-mature (56) or immature (57) DC (so-called DC10 or DC-10, respectively), express an array of inhibitory receptors (e.g., PD-L1, PD-L2, ILT3, ILT4, HLA-G) (56, 57). It has long been known that HLA-G-expressing antigen-presenting cells can induce T cell anergy (58), but it was more recently recognized that the levels at which HLA-G is expressed by DC-10, at least, correlates with the cell’s regulatory activities (59). However, not all inhibitory factors expressed by a DCreg necessarily contribute to the cell’s regulatory activities. Thus, vitamin D/IFNγ-induced human DCreg express IL-10, HLA-G, PD-L1 and low levels of FasL, but among these it was only their expression of PD-L1 that was reported as integral to the cell’s regulatory activities (60). It is also apparent that inhibitory receptors can play more than one role in immune tolerance. For example, the PD-L1 expressed by TGFβ-induced DCreg is important both to the induction of T cell apoptosis and the differentiation of Treg (61), both of which are essential to successful immunotherapeutic outcomes. Indeed, it has been suggested that the PD-L1:CD86 expression ratio within vitamin D/IL-10-induced DCreg will be a useful predictive marker of the cell’s immunotherapeutic efficacy in the clinic (62). Another, less-reported DC inhibitory receptor is CD31, expressed at high levels on GM-CSF-induced mouse bone marrow DC and on human CD34^+^ stem cell-differentiated DC that have been exposed to VitD. Engagement of CD31^+^ DC with T cells strongly inhibits their activation (63), at least in part by inducing rapid T cell disengagement from the DC, effectively raising their activation threshold [reviewed in ref (64)].

Regulatory DC are also well known for their soluble mediators that contribute importantly to their tolerogenic activities. For example, IL-10 is probably the most reported of the inhibitory signals emanating from DCreg (50, 65). It inhibits the antigen-presenting functions of DC (66), but also leads to its own upregulation in these cells. We know that IL-10 expression is essential to the tolerogenicity of IL-10-induced murine DC10 - silencing or deletion of its expression in these cells eliminates their regulatory activities (67, 68). Human and murine DC10 have been reported to suppress Th2 responses *in vitro* and *in vivo* (56, 68–70), perhaps related to IL-10-induced granzyme B expression in these T cells and thereby apoptosis (71). TGFβ secretion also contributes to immune tolerance. Its expression by tumor cells can foster the expression of IDO by endogenous tumor DC and thereby suppress anti-tumor immunity (72), but numerous populations of DCreg have been shown to also express TGFβ and to similarly suppress Teff cells [reviewed in (50)]. Vitamin D/dexamethasone-induced DCreg secretion of TGFβ suppresses both IFNγ production and proliferation of CD4^+^ T cells from rheumatoid arthritis patients (73), just as TGFβ produced by steady state CD8α^+^ DC or all-trans retinoic acid-induced DCreg (DC-RA) contributes to suppression of allergic donor Teff cell responses in mouse models (51, 74). TGFβ signaling dampens TCR-induced Ca^++^ influx in T cells, preventing their activation, but it also silences expression of the transcription factors T-bet and STAT4, which are critical to Th1 cell differentiation (72). IL-27 is another cytokine that is intimately linked to the induction of tolerance—it can reduce IL-2 expression during Th1 cell differentiation but also, when coupled with IL-6 signaling, can induce Th1, Th2, and Th17 cells to secrete IL-10 (75). We reported that the DC-RA noted above also secrete high levels of IL-27, but that neutralizing IL-27 does not affect the activation of Th2 cells from peanut allergic mice seen in co-cultures of DC-RA and allergen-presenting immunostimulatory DC. Rather, wild-type (w.t.) DC-RA fully protect against peanut-induced anaphylaxis *in vivo* by driving the differentiation of LAG3^+^CD49b^+^Maf^+^Foxp3^−^ Treg—unlike w.t. DC-RA, IL-27^-/-^ DC-RA are of no therapeutic benefit (74). IL-27 has also been reported to induce the differentiation of IL-10-secreting Tr1 cells through induction of c-Maf, IL-21 and ICOS in T cells [reviewed in (75)].

### DCreg Induce Differentiation of Regulatory T Cells

There are of course multiple populations of CD4^+^ Treg. CD25^+^Foxp3^+^ Treg, which are probably the most commonly reported Treg, include both naturally-occurring thymic emigrants [natural Treg (nTreg)] as well as cells induced to differentiate in the periphery (induced Treg) from either naïve (76) or effector (77) CD4^+^ T cells. Interestingly, when the activities of nTreg and DC10-induced Treg of identical TCR specificity were compared in a preclinical model of asthma, the latter cells carried markedly (i.e., ≈5-10-fold) greater regulatory activity than the nTreg (78). There are also inducible IL-10-dependent type-1 Treg (Tr1 cells) that are CD4^+^CD25^−^CD49b^+^LAG3^+^Foxp3^-^ (79), IL-10-independent CD25^+^CD49b^-^LAG3^+^Foxp3^−^ Treg (74), and oral tolerance-associated, TGFβ-dependent CD4^+^ Th3 cells (80).

While there is a large body of literature from preclinical models that confirms that DCreg can induce antigen-specific T cell tolerance *in vivo* (50, 65, 81–83), the collective literature addressing the function of human DCreg is less robust. Most studies with human DCreg have been restricted to showing that these DC only poorly activate allogeneic T cell responses *in vitro* (50). Fewer studies have assessed whether human DCreg suppress autologous Teff responses and induce Treg differentiation [e.g., (56)], or critically examined the phenotype and tolerogenic mechanisms of the induced Treg. This is an important issue, for expression of Treg markers in itself does not necessarily mean that the putative Treg are functional. A case in point is a recent report relating to the use of immature human DC-10 to induce tolerance among circulating T cells of peanut-allergic donors. Peanut allergen-presenting DC-10 did indeed induce the differentiation of T cells that expressed the expected Tr1 markers, but these cells lacked Tr1 cell activity, as determined in functional assays (84). Whether this could be related to the recently reported Th2 adjuvant activities of peanut proteins (85) has not been assessed as yet. However, we have shown that allergen-loaded semi-mature DC10 from grass- or cat-allergic donors both suppress Th2 responses and induce the differentiation of fully functional CD25^+^LAG3^+^CTLA4^+^Foxp3^+^ Treg (56).

As in many areas of health research, we have gained important insights into the disease processes and therapeutic approaches from lessons learned in animal models. For example, just as IL-10-induced human monocyte-derived DC [whether immature or semi-mature (56, 57)] are tolerogenic *in vitro*, murine bone marrow-derived DC10 are potently tolerogenic *in vitro* and in mouse models of, for example, asthma (68, 69, 77). The regulatory activities of murine DC10 are critically dependent on their expression of IL-10 (67, 68) but also to a lesser extent their expression of CD80 and CD86 (67), IDO (70) and CD40 (Dawicki et al., under review). Interestingly, they induce cognate Th2 cells with which they are cultured to proliferate, but in doing so these Teff cells transdifferentiate into highly effective CD25^+^Foxp3^+^ Treg (77, 78). In contrast, mature retinoic acid-induced DC (DC-RA) induce no proliferative responses among Th2 cells, although the T cells differentiate into potent LAG3^+^CD49b^-^Foxp3^-^ Treg (74). Thus, overall it is clear that numerous signals that DCreg can bring to interactions with naïve or effector T cells can induce these cells to take on a regulatory phenotype.

Regulatory T cells are activated in a T cell receptor-dependent fashion but, once activated, they are able to suppress the responses of by-stander T cells through a number of non-specific signals (e.g., secretion of IL-10) (86–91). However, bystander DC can similarly adopt a regulatory phenotype following interactions with Treg, and thereby further foster tolerance through a process known as infectious tolerance (92). Treg have a number of mechanisms by which they can induce DCreg, including CTLA-4 induction of IDO (88), LAG3 activation of inhibitory signals (93), or neuropilin-1 signaling (88). Expression of galectin-1 by Foxp3^+^ Treg (86) induces DC to secrete IL-10 and IL-27 (94), through which they can foster Tr1 cell differentiation (95). And Treg can themselves recruit additional populations of Treg without a need for a DC intermediary. For example, TGFβ released from Th3 cells triggers development of CD25^+^Foxp3^+^ Treg (96), while LAP-TGFβ on activated Foxp3^+^ Treg can induce naïve T cells to adopt a similar Foxp3^+^ Treg phenotype (97).

#### DCreg Production and Maturation

Currently, the most commonly used approach for generation of human DCreg includes differentiation of purified CD14^+^ peripheral blood monocytes into immature DC by culture with GM-CSF and IL-4 (98–100). Murine DCreg, thought to belong to the cDC2 category, are similarly differentiated with GM-CSF and IL-4, but most often from bone marrow progenitors (101). In principle, induced pluripotent stem cells could be used to generate very large numbers of DCreg (102–105), but use of such approaches has been tempered by concerns about the potential for pre-existing epigenetic programming in the stem cells and, more on moral grounds, the phenotypic similarities of stem cells to human foetal cells (106–109). All things considered, the use of mo-DCs is presently the industry standard for *in vitro* generation of human DCreg for clinical and experimental applications. Tolerogenic agents [reviewed in (50)] are added to the cultures of differentiating cells at varying times, depending on the type of DCreg in question. Because of the high likelihood that DCreg being used for immunotherapeutic purposes will be exposed to marked inflammatory signals after delivery to the recipient, it is critical that the treatment DCreg not convert into immunostimulatory cells that might exacerbate, rather than ameliorate pathology (110). As such, it is routine that DCreg are assessed for their abilities to withstand such phenotypic conversion following challenge with inflammatory mediators (e.g., IL-1β, IL-6, TNF) or TLR agonists [reviewed in (50)]. Maturational markers for DC include increased expression of HLA-DR, co-stimulatory molecules and inflammatory cytokines (e.g., IL-12) (33, 111–113). On the other hand, inflammation-resistant DCreg retain reduced expression levels of these maturational markers while maintaining their expression of inhibitory receptors and anti-inflammatory cytokine secretion, as noted above (44, 45, 73, 74, 94, 95, 114–117).

There are many well-established protocols to generate DCreg, but it is important to keep in mind that the populations of regulatory cells that we generate include both DCreg and, almost inevitably, additional sub-populations of monocyte-derived progeny that may or may not have their own activities. While some DCreg reports do include, for example, a final CD11c^+^ DC selection step (e.g., MACS- or FACS-sorting) prior to use of the DCreg generated, the majority of reports do not, suggesting that any effects observed may not be exclusively attributable to the DCreg. For example, a recent report of murine DCreg induction with varying doses of vitamin D (DC-VitD) revealed that there was a dose-dependent output of CD11c^+^ cells. The control immature DC in these experiments were ≈82% CD11c^+^, while the DC-VitD comprised up to 92% CD11c^+^ cells. Thus, while the “DC-VitD” pool of cells strongly suppressed CD4^+^ T cell activation, potential contributions to that activity of the ≥8% non-DC-VitD were not assessed (63). Depending on the precise culture conditions, culture of CD14^+^ human monocytes in GM-CSF and IL-4 can lead to differentiation of a mixed population of CD83^+^ DC, CD14^+^ macrophage-like cells, and/or myeloid-derived stem cells [reviewed in (118)]. Moreover, while CD83^+^ DCreg remain the predominant cell in these cultures, a proportion of the CD14^−^ cells present subsequently regain their expression of CD14 and their macrophage-like properties when exposed to IL-10 and maturational signals. The authors reported that the presence of even small numbers of these contaminating CD14^+^ macrophage-like cells skewed the apparent regulatory phenotype of the “DCreg population” in a dose-dependent fashion (118). It has also been reported that human DC10 generated from plastic-adherent monocytes comprise two major sub-populations, including CD83^hi^HLA-DR^hi^CCR7^+^ cells that strongly express CD25, and CD83^lo^HLA-DR^lo^CCR7^lo^ cells. Both populations can suppress Teff cell proliferation and induce Treg differentiation, but the CD83^hi^HLA-DR^hi^ population is significantly better in both tasks, at least in part because the regulatory activities in these DC10 cultures were CD25-dependent (119). Taken together, these outcomes do not diminish the importance of DCreg, but they do raise the question of whether contaminating cells in, for example, monocyte-derived cultures might also contribute significantly, but in a negative manner, to the regulatory outcomes observed. This would be relevant for functional studies, but it would be critical to have such insights when undertaking, for example, global transcriptomic studies of DCreg.

#### DCreg Control of Tolerance Responses

In order for DCreg to be clinically relevant, the protocols used for their induction must be optimized, keeping in mind the intended target disease. This is in part because the types of infectious tolerance processes launched by the chosen DCreg must fit the clinical indication. For example, as suggested above, both DC-VitD/dex and DC10 secrete IL-10 and thereby induce Th2 cells to differentiate into CD25^+^Foxp3^+^ Treg which can fully reverse the asthma phenotype (68, 69, 77, 120). On the other hand, as noted above DC-RA production of IL-10, TGFβ, and IL-27 suppresses peanut anaphylaxis-inducing Th2 cells through induction of LAG3^+^CD49b^−^Foxp3^−^ Treg (74). The relevance of this distinction lies in the observation that intestinal inflammation can suppress Foxp3 expression in a mouse model of colitis, such that intestinal Foxp3^+^ Treg therein convert into pathogenic Th17 cells (121). This begs the question of whether use of DCreg strategies that induce Foxp3^−^ Treg, which could be more inflammation-resistant than the Foxp3^+^ Treg, would be better suited in this context. Similar inflammation-associated adverse outcomes have been reported in other DCreg immunotherapy models (122, 123). Having said that, it has also been reported that CD40/CD80/CD86-knock down DCreg also induce the differentiation of Foxp3^+^ Treg in a murine colitis model, but that this treatment is successful in preventing leukocyte infiltration and disease development (124). This indicates that, while it is critical that they be taken into consideration, inflammatory conditions that may be seen by treatment DCreg *in vivo* do not necessarily lead to adverse outcomes, and that bodes well for DCreg immunotherapeutics.

DCreg routinely recruit Treg into infectious tolerance processes, but induction of Treg is not an essential facet of DCreg-induced tolerance. For example, it has been reported that human vitamin D-induced DCreg foster allogeneic T cell differentiation into classical Treg (125–128), but also that at least some forms of vitamin D-induced DCreg can instead foster a Treg-independent type of tolerance. Thus, murine CD11c^+^ DC that had been differentiated in GM-CSF and vitamin D, or human DCreg differentiated from CD34^+^ stem cell precursors with vitamin D do not induce Treg responses, but instead impair CD4^+^ T cell priming in a CD31-dependent fashion (63). Silencing of CD31 increases T cell activation in DCreg: T cell co-cultures, while its overexpression leads to substantially reduced DC:T cell contact times, with reductions in IL-2 production by the T cells and a consequent loss of T cell priming (63). Collectively this evidence suggests that we must be cognizant that even seemingly closely-related populations of DCreg may utilize quite disparate regulatory mechanisms, and that should be taken into consideration when planning immunotherapeutic approaches. Although such variance may be difficult to predict, there are emerging trends in the published literature.

**Tables 1** and **2** outline our more recent advances in the biology of non-human (murine, NHP) and human DCreg, respectively. Because we comprehensively reviewed this area previously (50), these tables provide only an update on observations regarding novel agents to induce the DCreg, or the use of previously reported DCreg in new models, rather than a comprehensive listing of all types of DCreg investigated to date. Thus, the interested reader can find additional information on human DCreg found across our organ systems and those induced *in vitro* with IL-10 (6, 56, 57, 68–70, 153–161) or other cytokines (160–165), corticosteroids (156, 166–168), vitamin D3 (127, 167, 169–172), rapamycin (156, 167, 173, 174), and neuropeptides (175, 176) in the references cited. **Table 1** provides *in vivo* data from animal models of human diseases, and includes the agents used to induce and mature murine and NHP DCreg, the disease model addressed, the phenotype of the DCreg and the mechanisms by which it induces tolerance, its clinical effects in that model and whether it activates secondary regulatory processes (e.g., Treg).

**Table 1:** Studies examining induced non-human regulatory DC.

Induction Agent (Mat. Agent)	Species (Admin. Route)	Disease Model	DC Phenotype	Mechanism of Tolerance	Performance Outcomes	Secondary Induction of Tolerance	Reference
**Murine models**							
**9-cis-retinoic acid** **(LPS)**	Mouse (s.c.)	Contact hyper-sensitivity	↓ CD103, CD207, MHCII, CD80, CD86 ↑ PD-L1, osteopontin	Osteopontin-dependent	↓ contact hyper-sensitivity	↑ CD25^+^ Foxp3^+^ Treg	(129)
**all-trans-retinoic acid** **(LPS)**	Mouse (i.p.)	Food (peanut, egg) allergies	↑ MHCII, TGFβ, Aldh1A2, PD-L2, IL-27	Th2 anergy, IL-10 & TGFβ-dependent; Treg induction, IL-27-dependent	↓ anaphylactic responses	↑ CD25^+^ LAG3^+^CD49b^−^ Foxp3^−^ Treg	(74)
**Allograft inhibitory factor-silenced DC** **(LPS)**	Mouse (s.c.)	OTII TCR-transgenic T cell responses *in vivo*	No discernible changes in DC marker or cytokine secretion vs. w.t. DC-LPS	–	↓ T cell proliferation & IL-2, IFNγ & TNF secretion	↑ CD4^+^CD25^+^ Foxp3^+^ Treg	(130)
**c-reactive protein** **(LPS)**	Mouse (N/A)	EAE	↓ MHCII/CD86/CD40 ↓ CD11b+CD11c+BMDC ↓ IL-10/IL-12p70 production	FcγRIIB-dependent	↓ Antigen-specific T cell proliferation	–	(131)
**CD40 siRNA-silenced**	Mouse (i.p., i.n., i.v., or s.c.)	Allergic Rhinitus	↓ IL-4/IL-5 production ↑ IL-10, IL-35 production	N.D.	↓ symptoms, & tissue IL-4, IL-5 & eosinophils (i.n. DCreg only)	↑ IL-10, IL-35 & FoxP3 expression	(132)
**CD40/80/86-silenced DC** **(N/A)**	Mouse (i.p.)	Colitis	–	–	Prevention of colitis development ↓ Leukocyte infiltration to colon	↑ FoxP3^+^ Treg ↑ IL-10^+^ Breg	(124)
**Dexamethasone** **(LPS)**	Mouse (i.p.)	chronic Chagas disease cardio-myopathy	↓ MHCII/CD86/CD80/CD40 ↓ IL-6/IL-12 ↑ IL-10 production	–	↓ Heart inflammation & fibrosis ↓ IL-2/IFNγ *in vitro*	↑ CD4^+^FoxP3^+^ Treg	(133)
**Dexamethasone** **(LPS)**	Mouse (i.p.)	Dust Mite Allergy	↓ MHCII/CD86/CD80/PD-L2 ↓ IL-6/IL-12 ↑ IL-10 production	–	↓ Inflammatory cells in lungs and mucus deposition ↓ Antigen-specific IgE ↓ *Gata3* & *IL-4* in lungs	↑ FoxP3^+^ Treg	(134)
**Dexamethasone** **(IFN-γ & LPS)**	Rat (N/A)	–	↓ MHCII/CD86/CD80/IL-6/IL-12 ↓ Inducible NO synthase ↑ Sialic acid upregulation	Partially sialic acid-dependant	↓ allogeneic T cell proliferation & activation	–	(135)
**Minocycline** **(IFN-γ & TNF-α or LPS)**	Mouse (i.v.)	EAE	↓ MHCII/CD54/CD80/CD86/ IL-1β/IL-6/IL-12/TNF ↑ PD-L1	–	↓ Allogeneic T cell priming ↓ EAE clinical responses	↑ CD4^+^ CD25^+^FoxP3^+^	(136)
**Minocycline & Dexamethasone (IFN-γ & TNF-α or LPS)**	Mouse (i.v.)	EAE	↓ MHCII/CD54/CD80/CD86/ IL-1β/IL-6/IL-12/TNF	Partially IL-10-dependant	↓ Antigen-specific T cell proliferation ↓ Clinical responses	↑ FoxP3^+^ Treg	(137)
**IL-10**	Mouse (i.p., s.c., i.v., t.t.)	Asthma	↓ MHCII/CD80/CD86/IL-12 ↑ IL-10	↓ Th2 response. ↓ B cell response. - Convert Teff to CD25^+^Foxp3^+^ Treg - IL-10-dependent	i.p. or t.t. eliminate asthma phenotype s.c. ↓ Th2 response	↑ FoxP3^+^ Treg	(68–70, 77)
**Lentiviral- IL-10 upregulation** **(LPS)**	Mouse (i.v.)	GVHD	↑ IL-10 production	–	↓ *In vitro* T cell proliferation & activation ↑ Survival times ↓ GVHD scores	↑ CD4^+^CD25^+^ LAG3^+^CD49b^+^FoxP3^−^ Tr1 cells ↑ IL-10, IFN-γ	(138)
**RelB-silencing** **(LPS or CpG-B DNA & CD40L)**	Mouse (N/A)	SLE	↓ MHCII/CD40/CD86 ↓ IL-12/TNF	–	↓ Ag-specific splenic T cell activation ↑ IL-10 production ↓ IFN-γ/IL-17 production	Possible FoxP3^−^ IL-10-producing Treg	(139)
**METTL3-silencing** **(LPS)**	Mouse (i.v.)	Heart transplant	↓ MHCII/CD80/CD86 ↓ IL-12/IFN-γ ↑ IL-4/10 production	–	↓ Th1:Th2 ratio ↓ Survival times ↓ pathological scores	–	(140)
**Vitamin D & Dexamethasone (LPS)**	Mouse	proteoglycan-induced Arthritis	↓ MHCII ↓ PD-L1/CD86 ratio ↓ IL-10 production	–	↓ Antigen-specific T cell proliferation ↓ Clinical responses	↑ FoxP3^+^ Treg	(141)
**NON-HUMAN PRIMATE**							
***VitD3* & IL-10** **(TNF/IL-1/IL-6)**	Rhesus macaque (i.v.)	Renal transplant	↑ HLA-DR/CD86 ↑ PD-L1	↓ Allo-T cell proliferation ↓ IL-17 production	↑ graft survival times (NS)	↑ CD4^+^ CD25^+^FoxP3^+^ CTLA4^hi^ Treg	(141–146)

Mat. Agent, maturation agent; Admin. route, administration route; LPS, lipopolysaccharide; EAE, experimental autoimmune encephalomyelitis; BMDC, bone marrow-derived DC; Breg, regulatory B cell; NO, nitric oxide; GVHD, graft-versus-host disease; SLE, systemic lupus erythematosus; METTL3, methyltransferase-like protein 3; VitD3, 1,25-dihydroxyvitamin D3.

**Table 2:** Studies examining induced human regulatory DC.

Induction Agent (Mat. Agent)	DC Phenotype	Mechanism of Tolerance	Regulatory effects	Secondary Induction of Tolerance	Ref
**Dexamethasone/*VitD3* (LPS)**	↓ CD83/CD86/CD40	↓ Akt and p38MAPK protein phosphorylation	↓ Autologous T cell proliferation ↓ Th1/Th17 Differentiation	↑ FoxP3^+^ IL-10-secreting Treg	(147)
**Dexamethasone/*VitD3*** **(LPS)**	↓ CD80/CD83/CD86 ↑TGF-β1 production	Partially TGF-β1-dependant	↓ Allogeneic CD4^+^ T cell proliferation ↓ IFN-γ production	–	(73)
**Ethyl pyruvate** **(TNF, IL-1, PGE2)**	↓ CD40/CD83/CD86/HLA-DR ↓ IL-6, IL-12, TNF-α production	–	↓ Allogeneic CD4^+^ T cell proliferation ↓ IL-17/IFN-γ production	–	(148)
**IL-10** **(TNF, IL-6, IL1, PGE2)**	Two DC sub-populations: **i -** CD83^hi^CCR7^+^HLA-DR^hi^ ↑ CD25 (membrane & soluble) **ii -** CD83^lo^CCR7^-^HLA-DR^lo^ Lower CD25	Largely CD25-dependant	↓ Allogeneic CD4^+^ T cell proliferation with both CD83^hi^ HLA-DR^hi^ > CD83^lo^HLA-DR^lo^ cells	CD83^hi^ HLA-DR^hi^-induced Treg more potent than CD83^lo^HLA-DR^lo^-induced Treg	(119)
**IL-10-lentivirus** **(TLR agonists or TNF-α, IL1β and IL-6)**	↑ CD14/CD16/CD141/ CD163/HLA-DR ↑ ILT4/HLA-G ↓ CD1a ↑ IL-10 production	–	↓ Allogeneic CD4^+^ & CD8^+^ T cell proliferation ** _____** NSG mouse model: ↓ Severity of GVHD ↑ GVHD survival time	↑ Treg (Tr1)	(149)
**Retinoic acid** **(LPS)**	↓ CD80/CD86/ HLA-DR ↑ CD141, GARP	CD141 & GARP-dependent	↑ Allogeneic CD4^+^ T cell IL-10	↑ CD4^+^CD25^+^ FoxP3^+^ Treg	(150)
**Staph peptides** **(LPS or staph cell lysate)**	↓ CD40/CD80/HLA-DR ↓ IL-10, IL-12, TNF-α	Possibly ↓ NF-κB and p38 pathway phosphorylation	↓ Antigen uptake by DC ↓ autologous Th1 priming ↓ IFN-γ production ↑ IL-13 production	↑ CD4^+^ CD127^-^ CD25^hi^ CD45RA^-^ FoxP3^hi^ iTreg	(151)
***VitD3*** **(IFN-γ & LPS)**	↓ HLA-DR/CD86/CD80 ↑ PD-L1/ILT-3/CD52 ↓ IL-10/TNFα/IL-12 production	–	↓ allogeneic T cell proliferation *in vitro* ** _____** NSG mouse model: ↓ Severity of GVHD with autologous PBMC reconstitution	↑ FoxP3^+^ Treg	(125)
***VitD3* (+/-** **manipulation of CD31)** **(MBP +/- LPS)**	-	↑ CD31 expression	↓ Autologous CD4^+^ T cell priming ↓ CD4^+^ T cell interaction time ↓ IL-2/IFN-γ/GM-CSF/TNFα production	–	(152)
***VitD3* & IL-10** **(LPS, CD40L or TNF/IL-6/IL-1β/PGE2)**	↓ CD80/CD86 ↑ PD-L1 ↑ IL-10 & IL-12 production	Partially PD-L1-dependant	↓ Allogeneic CD4^+^/CD8^+^ T cell proliferation ↓ IFN-γ/IL-17a/perforin /granzyme B production	–	(62)

Mat. Agent, maturation agent; VitD3, 1,25-dihydroxyvitamin D3; LPS, lipopolysaccharide; GVHD, graft-versus-host disease; PGE2, prostaglandin E2.

**Table 2** provides data from human DCreg generated from CD14^+^ monocytes *in vitro*, and includes the agents used to induce and mature the cells, the phenotype of the DCreg, the mechanisms by which they induce their immunologic effects and whether they activate Treg. While the regulatory activities for most of these cells were assessed exclusively *in vitro*, the IL-10-lentivirus-tranfected human DC10 were also assessed for their abilities to protect humanized otherwise immunocompromised mice from graft-versus-host disease (149).

#### Impact of Microbial Exposures on the Phenotype of DCreg

There are a number of questions that should be addressed before DCreg immunotherapy can become mainstream as a clinical approach. As suggested above, a particularly important one is the nature of inflammatory conditions the DCreg may face after delivery to the host and their impact on the cell’s regulatory activities. We have previously reviewed the array of innate immune receptors expressed by DC, which include protease-activated receptors (PARs), TLR, C-type lectin receptors, retinoic acid-inducible gene-1 (RIG-1), and the melanoma differentiation-associated gene-5 (MDA-5) (50), but they also express receptors for an array of other inflammatory mediators (177, 178). As noted previously, there are multiple reports that the tolerogenic potential of DCreg can be arrested, or even reversed, under inflammatory conditions, such that the cells instead activate effector T cells and thereby exacerbate pathology (122, 123, 179). Despite the fact that many microbial stimuli have adverse effects on DCreg, there is an accumulating body of evidence that other microbial signals contribute importantly to the regulatory activities of these cells. For example, delivery of the TLR5 agonist flagellin to asthmatic mice ameliorates their disease phenotype through induction of DCreg and, subsequently, Treg responses (180). Moreover, exposure of monocyte-derived DC from house dust mite (HDM)-asthmatic individuals to flagellin also empowers the DC to take on a regulatory phenotype. These flagellin-induced DCreg express high levels of IL-10, TGFβ and HLA-G, such that they strongly foster CD25^+^Foxp3^+^ Treg responses (180). In a similar manner, delivery of the dectin-1/TLR2 agonist curdlan (181), a bacterial cell wall exopolysaccharide, to asthmatic mice induces differentiation of IL-10-expressing, Maf^+^Foxp3^−^ Treg (i.e., Tr1 cells), and thereby reverses the animal’s asthma phenotype (182). On the other hand, human IL-10-lentivirus-transfected DCreg display very complex responses to microbial agonists (149), including polyinosinic:polycytidylic acid (Poly I:C), LPS, flagellin and CpG (agonists specific for TLR3, TLR4, TLR5, and TLR9, respectively), as well as *Listeria monocytogenes* (149). The authors of this report found that a number of classical DCreg markers were differentially regulated on exposure to these agonists. For example, poly I:C challenge increased HLA-G and decreased ILT4 expression, while LPS exposure down-regulated HLA-G and up-regulated ILT4. In contrast to IL-10-lentivirus-transfected murine DCreg, which induce differentiation of CD25^+^Foxp3^+^ Treg (159), the IL-10-lentivirus-transfected human DCreg instead induced the development of Tr1 responses (149). Interestingly, however, while the numbers of Tr1 marker-positive cells were differentially affected by the microbial agonists added to the human DCreg-T cell cultures, marked differences in the suppressive activities or cytokine profiles of the Tr1 cells so induced were not observed (149).

The recognition that our microbiome contributes importantly to our overall health has brought its impact on immune regulation by DC into focus. We know, for example, that the diversity of the microbiome in neonates of ≤100 days of age can predict their likelihood of developing asthma as children (183). Reduced levels of *Lachnospira*, *Veillonella*, *Faecalibacterium*, and *Rothia* genera bacteria in the neonatal gut are associated with an increased risk of asthma. The neonates so affected display reduced levels of fecal acetate, but see increased urinary excretion of a number of secondary bile acids with potential links to this intestinal dysbiosis (183). We know that microbial catabolites (e.g., short-chain fatty acids) such as butyrate and propionate can induce a tolerogenic phenotype in DC, dampening LPS-induced expression of costimulatory molecules (e.g., CD83, CD40) and augmenting downstream Tr1 responses (184). And culture with *Lactobacillus paracasei* L9 induces murine bone marrow-derived DC to strongly upregulate IL-10 secretion and to take on a regulatory phenotype, such that they induce CD4^+^ effector T cells from β-lactoglobulin-allergic mice to differentiate into Foxp3^+^ Treg (185). Similarly, in experimental germ-free mice β-glucan/galactan polysaccharides and polysaccharide A secreted by intestinal *Bifidobacterium bifidum* strain PRI1 are associated with increased expression of IL-10, TGFβ, IDO, and PD-L2 within the lamina propria CD103^+^ DC population (186). Importantly, these DC in turn induce the development of Treg with TCR specificity not only for *B. bifidum*, but also for otherwise unrelated intestinal antigens (186). And *Bifidobacterium infantis* colonization has been reported to induce shrimp tropomyosin tolerance in a mouse model of shrimp allergy, increasing the gut population of CD103^+^ DCreg, expression of IL-10 and TGFβ, and the numbers of CD4^+^CD25^+^CD127^-^ Treg (187).

Unlike pathogenic enteric bacteria, which actively secrete pathogenic mediators within the gut, tolerance-promoting gut symbionts reportedly interact with their hosts *via* capsular polysaccharides that decorate outer membrane vesicles (OMV) shed by the bacteria. Thus, *Bacteroides fragilis* OMV signal to gut DC through TLR2, which induces the development of DCreg and Treg (188), but there can be heterogeneity in the way that gut DC respond to such signaling. Thus, while *Bacteroides thetaiotaomicron* OMVs induce IL-10 expression and a regulatory phenotype in colonic DC of healthy subjects, such immune regulation does not occur with DC from individuals with ulcerative colitis (189). Organisms with beneficial properties can also be found in the external environment. Indeed, curdlan, the tolerogenic β-glucan noted above (182), is derived from the environmental bacterium *Alcaligenes faecalis.* In a similar manner, exopolysaccharides from *Cyanobacterium aponinum*, an organism represented at high levels in the waters of the Blue Lagoon of Iceland, induce human DC to express IL-10 and CD141, leading to Treg induction (190, 191). Bathing in the Blue Lagoon is renowned for its abilities to alleviate psoriatic plaques (191).

#### Regulatory DC for Clinical Trials

There have been scores of preclinical studies in mouse models documenting that DCreg can induce immunologic tolerance and reverse the disease phenotype in models of allergy and asthma, autoimmune diseases and transplant rejection responses [reviewed in (50)]. As part of moving DCreg closer to clinical application there have also been a number of DCreg trials undertaken in NHP models (**Table 1**). An early study with maturation-resistant VitD3/IL-10-induced rhesus macaque DCreg showed that cells given i.v. led to an initial increase in recipient blood levels of anti-donor and -third party T cell reactivity, but this subsequently waned to below pre-treatment levels in animals also treated with the clinical immunosuppresant CTLA4Ig. There was no induction of anti-donor IgG or IgM alloantibodies detected and, in animals given both DCreg and CTLA4Ig, alloreactive IL-10-producing T cells were detected at ≥28 days post-infusion (143). Culture of such VitD/IL-10-induced NHP DCreg with purified allogeneic peripheral blood CD4^+^CD127^lo^ cells, which were enriched in Foxp3-expressing cells (i.e., Treg), for up to 2 weeks led to an expansion of the CD4^+^CD127^lo^ Treg, and these in turn induced a dose-dependent suppression of CD4^+^ Teff cell responses (144). A subsequent study with kidney-engrafted rhesus macaque monkeys treated with donor-derived VitD3/IL-10 DCreg showed that the DCreg significantly prolonged graft survival (from ≈39 to 113 dy) and reduced donor-reactive memory T cell:regulatory T cell ratios, with no evidence of circulating donor-specific alloantibodies (146). In another study, VitD/IL-10-induced kidney transplant recipient DCreg were pulsed with donor-derived PBMC membrane vesicles and used to treat transplanted monkeys that were also administered CTLA4Ig and rapamycin. Overall, the authors observed ≈2-fold-prolonged graft rejection times, attenuated systemic IL-17 levels, and modulated CD4^+^ and CD8^+^ T cell responses to donor antigens, although these were not statistically significant effects (142). The most recent NHP study reported addressed the impact of VitD/IL-10 DCreg treatment on CD4^+^CTLA4^hi^ (i.e., Treg) in CTLA4Ig-treated renal allo-transplant recipients. They found that in the absence of DCreg therapy, CTLA4Ig treatment led to reductions in the numbers of circulating CD4^+^CTLA4^hi^ Treg, while these reductions were not observed in DCreg-treated recipients (145). Overall, these studies highlighted the potential utility of DCreg immunotherapy in preventing rejection of organ transplants [reviewed in (192)].

Consistent with the outcomes of these NHP DCreg studies, there have been a number of successful Phase I or I/II human DCreg clinical trials completed by pioneers in this area in recent years (**Table 3**). There are reportedly additional trials that are still in recruitment stages or that have not yet published their results (200), as well as several long-term trials ongoing in organ transplant recipients [reviewed in ref (192)]. As these have been largely Phase 1 or I/II trials, their aims were solely to determine whether the DCreg immunotherapy approach in question is safe and well-tolerated, and it is clear that that is the case—the vast majority of subjects have not suffered significant adverse events (193, 195–199). Although, as might be expected for such trials, abrogation of disease was not seen under any of these protocols, reductions in symptoms scores were reported in a small subset of treatment group subjects in the rheumatoid arthritis (195, 197) and Crohn’s disease (196) trials. The observed changes in immunologic parameters in the treatment groups across these studies were encouraging, given that these were Phase I studies, though quite modest (**Table 3**).

**Table 3:** Human DCreg clinical trials.

Induction Agent (Mat. agent)	Status (n)	Disease target	DC-loading antigen	Method of DC Administration (Final Dose)	Performance Outcomes	Clinical Outcomes	Reference
**i. CD40/CD80/CD86-silenced** **ii. control DC** **(none)**	Phase 1; Completed (n = 10)	Type 1 diabetes	Autologous -	Intradermal (4 × 10^7^)	↑ B220^+^CD11c^−^ B cells in both control and exp groups	No adverse events, no clinical changes	(193)
**Semi-mature DC with rheumatoid antigens**	Phase 1; Completed (n = 12)	Rheumatoid arhtritis	PAD4, RA33, citrullinated-filaggrin, vimentin	Subcutaneous (5 doses each: low dose, 5 × 10^6^; high dose, 1.5 × 10^6^)	↓ IFN^+^ T cells & autoantibodies EULAR response, ↓ 33% to 83% (low vs. high dose)	C dose-dep effects (58% of subj had reduced RA scores)	(194) abstract only
**NF-κB inhibitor** **(N/A)**	Phase 1; Completed (n = 34; 16 control)	Rheumatoid arthritis	Citrullinated Peptides (4)	Intradermally (7.2 × 10^3^ – 6.2 × 10^4^)	↑ Treg:Teff cell ratios ↓ Serum IL-15, IL-29, CX3CL1 and CXCL11 ↓ T cell IL-6	Reduction of disease symptoms within 1 month of treatment	(195)
**Dex/Vit A** **(IL-1β, IL-6, TNF-α, & PGE2)**	Phase 1; Completed (n = 9)	Crohn’s Disease	–	Intraperitoneal (2/5/10 × 10^6^ or 6/15/30 × 10^6^)	↓ IFNγ (FACS) ↑ blood Th17 & Foxp3^+^ T cells	Reduction of disease severity within 12 weeks of treatment	(196) -
**Dex/VitD3 (MPLA)**	Phase 1; Completed (n = 13)	Rheumatoid arthritis	Autologous synovial fluid	Intra-articular (1/3/10 × 10^6^)	–	Reduction of disease symptoms for 2 patients	(197)
**Dex** **(IL-1β, IL-6, TNF-α, & PGE2)**	Phase 1b; Completed (n = 8)	Multiple Sclerosis	Myelin Peptides (7)	Intravenous (50/100/150 /300 × 10^6^)	↓ CD8^+^ & NK cells ↑ PBMC IL-10	–	(198)
**Dex** **(IL-1β, IL-6, TNF-α, & PGE2)**	Phase 1b; Completed (n = 4)	NMOSDs	Myelin Peptides (7) + AQP4	Intravenous (50/100/150 /300 × 10^6^)	↑ PBMC IL-10 ↓ *In vitro* antigen-specific T cell proliferation	–	(198)
**Dex/IL-10**	Phase I (n = 15)	Liver transplant	Donor-autologous	Intravenous (2.5–10 × 10^6^/kg)	↓ T-bet^+^Eomes^+^CD8^+^ T cells ↑ Foxp3^+^ Treg: T-bet^+^Eomes^+^ CD8 T cell ratios (pre-transplant)	No adverse reactions to DCreg infusion	(199)

AQP4, aquaporin-4; Dex, dexamethasone; EULAR, European League Against Rheumatism; Mat. agent, maturation agent; MPLA, monophosphoryl lipid A; NMOSDs, neuromyelitis optica spectrum disorders; PGE2, prostaglandin E2; Vit A, vitamin A; VitD3, 1,25-dihydroxyvitamin D3.

Human monocyte-derived DC are, overall, HLA-DR^+^CD11c^+^BDCA3^−^ and express an array of other prototypical DC markers (28) but there is substantial heterogeneity in the markers expressed by, and activities associated with any one DC population. Even seemingly very small differences in the DC culture conditions, from how the starting CD14^+^ monocyte population is isolated and its purity, to the concentrations of GM-CSF or IL-4 employed, the type of serum or serum-free supplement used in the cultures, times in culture, etc., can have very significant effects on the DC generated. This raises the academic question of whether differentiated tissue DC (e.g., blood, tonsillar) might be better candidate cells for DCreg immunotherapeutics—arguably their use could call for fewer investigator manipulations of the cells. Nevertheless, global transcript profiling of monocyte-derived DC, a number of DC cell lines and purified tissue DC reveals that there is substantial phenotypic heterogeneity here too. Monocyte-derived cells cluster in principle component analyses closely with tonsillar DC, but not other populations, while the blood DC populations tend to loosely cluster by themselves (201). However, developing a DCreg therapy using primary populations of DC would bring its own challenges, not the least of which is the relative scarcity of the cells. Fully differentiated blood DC constitute only 0.1%–1.0% of peripheral blood mononuclear cells (9, 202), such that it would not likely be feasible to purify sufficient numbers of primary cells from any one donor for an effective treatment. Thus, at this point in time, use of well-characterized and standardized monocyte-derived populations of DCreg would appear to be our best clinical option.

One of the more important factors in optimizing DCreg immunotherapy is identification of DCreg of a phenotype that best fits the intended application. There have been a number of reports that have run head-to-head comparisons of human DCreg differentiated with an array of mediators, including comparisons of vitamin D3-, dexamethasone- and rapamycin-induced cells (167), or of vitamin D3-, IL-10-, dexamethasone-, TGFβ− and rapamycin-induced DCreg (156), and assessments of DCreg differentiated with protein kinase C inhibitor versus dexamethasone, vitamin D3, rapamycin, IL-10, TGFβ or a combination of peroxisome proliferator-activated receptor gamma (PPAR-γ) agonist and retinoic acid (203). This has provided important insights into DC yields & viabilities, expression of co-stimulatory or inhibitory markers, IL-10 production, resistance to inflammatory stimuli, and abilities to activate allogeneic T cells or induce Treg (156, 167). Nevertheless, multiple questions remain even given our knowledge and advances in DCreg immunotherapy. While it is easy to appreciate the simplicity of using allogeneic cells for functional readouts with human DCreg, we question whether assessing their impact on autologous, disease-related T cells would more directly address the question of the cell’s suitability for immunotherapeutic applications (i.e., other than in the context of allogeneic transplantation). That is, given that the *raison d’etre* for DCreg therapy is more often than not suppression of specific antigen-driven pathology, would assessing the cell’s abilities to suppress autologous cognate T cell responses be more relevant (197)? Is assessing expression of Treg-associated markers (e.g., Foxp3) adequate to conclude that the putative Treg induced by these DCreg treatments are functional as regulatory cells? As noted above, at least some DCreg can induce differentiation of T cells that express Treg markers as determined by FACS, despite the fact that they are not functional regulatory cells (84). And, given that different DCreg use different strategies to induce tolerance (204) and thereby induce distinct types of Treg (50), should the nature of the condition being treated (e.g., low versus high levels of target tissue inflammation) impact the choice of the DCreg to be employed? As alluded to above, are there indications that we should target selective induction of Foxp3^+^ versus Foxp3^−^ Treg or, for that matter, CD4^+^ versus CD8^+^ or other regulatory cells? Another question is whether *in vitro* assays of DCreg function provide sufficiently robust data on the activities of these cells to validate them as candidates for clinical trials. Could *in vivo* modeling in, for example, humanized mice (205) or NHP (142–146) provide more meaningful insights?

While we often assess a standard set of markers associated with DCreg, it is clear that functional DCreg are more than cells that express low levels of CD40 or CD86, or high levels of PD-1 and IL-10. Indeed, numerous transcriptomic analyses have identified many hundreds of markers that are differentially expressed by DCreg (170, 206–210) but only three immunologically-relevant genes [annexin A1, glucocorticoid-induced leucine zipper (GILZ) and IDO] have been reported to display differential increases in expression within the DCreg across five or more of these reports, while several more (cathepsins B, C, D & L, ILT3, stabilin 1, and TGFβ) saw increases across three of these reports (211). Nevertheless, it is interesting that IL-10 expression, as an example, has not been so identified, particularly given its intimate association with DCreg across a spectrum of reports [reviewed in (50, 65)]. Are there other leads we are missing in this search? Examination of the blood DC from allergic individuals who have undergone allergen-specific immunotherapy have identified a number of markers associated with successful tolerance induction (e.g., C1q, stabilin-1) (210). Indeed, administration of C1q in mouse models of asthma was subsequently shown to also effectively reduce the asthma phenotype in these animals (212). Intuitively, it seems that each population of DCreg likely enjoys contributions from a large number of regulation-associated inputs (e.g., expression of inhibitory mediators and receptors, low levels of HLA-DR and co-stimulation, etc.) that cumulatively define the cell’s regulatory activities, with different populations of DCreg employing distinct levels of large numbers of these as unique inputs.

## Conclusion

Currently, the standard of care for allergies and autoimmune disorders comprises the use of immunosuppressive drugs, agents which often have substantial deleterious side-effects and must be administered for the life of the patient. Regulatory dendritic cell research is promising in that DCreg-induced immune regulation appears to be robust and enduring (70), at least as far as we have been able to ascertain in animal models. Murine DCreg treatments can reverse or eliminate even severe disease in models of allergies and autoimmune diseases and extend the life of organ transplants, sometimes indefinitely. They do so by activating infectious tolerance processes that incorporate secondary induction of different types of Treg which, in turn, can recruit additional generations of regulatory cells. Though animal model outcomes cannot be directly translated to human conditions, the burgeoning *in vitro* research and early-stage clinical trial outcomes with human DCreg bode well for their use in the clinic. Certainly, our DCreg clinical trials to date indicate that DCreg immunotherapy is safe and well-tolerated. Our increasing advances in the cellular and molecular biology of these cells is likely to have a significant impact on the efficacy of DCreg in the clinic in the foreseeable future. Unlike our current pharmacological management of these diseases, immunotherapy with DCreg would seem to allow for the possibility of long-term restoration of the physiologic equilibrium associated with immunologic tolerance.

## Author Contributions

SN wrote the first drafts of the manuscript. SL contributed to the literature searches. JG contributed to the writing and edited the manuscript. All authors contributed to the article and approved the submitted version.

## Funding

Funding was provided by grants from the Canadian Institutes of Health Research (MOP53167) and the AllerGen Networks of Centers of Excellence (16B&B10).

## Conflict of Interest

The authors declare that the research was conducted in the absence of any commercial or financial relationships that could be construed as a potential conflict of interest.
